# Alcohol Beverage Household Expenditure, Taxation and Government Revenues in Broader European WHO Region

**DOI:** 10.3389/fphar.2017.00303

**Published:** 2017-05-26

**Authors:** Mihajlo Jakovljevic, Elena A. Varavikova, Henriette Walter, Alexander Wascher, Ana V. Pejcic, Otto M. Lesch

**Affiliations:** ^1^Health Economics and Pharmacoeconomics, Faculty of Medical Sciences, University of KragujevacKragujevac, Serbia; ^2^Federal Research Institute for Public Health Organization and Information (CNIIOIZ), Ministry of HealthMoscow, Russia; ^3^Department of Psychiatry and Psychotherapy, Medical University of ViennaVienna, Austria; ^4^Yeshiva UniversityNew York, NY, USA; ^5^Faculty of Medical Sciences, University of KragujevacKragujevac, Serbia

**Keywords:** alcohol, household expenditure, taxation, government revenues, Europe

## Global industry of alcoholic beverages and its outreach

The alcohol beverage industry became a significant source of income for several branches in the economy (Kasim, 2006). This has become highly visible in Third World countries during recent years (Caetano and Laranjeira, 2006). Although the alcohol beverage producing industry played an important role in the development of Western economies, its long term balance of gains and harms remains highly disputable (Room and Jernigan, 2000). Sales trends seem to be heavily affected by accelerated globalization. A good example for this is the fact that one of the major manufacturers, Diageo®, which owned 83% of the sales to traditional North American and Western European consumers. However, in 2016 this share has fallen dramatically to only 42% (Institute of Alcohol Studies, 2016). The same scenario has been applied to all top producers such as SABMiller®, Heineken®, Karlsberg®, and others. All of those redirecting their long term investment and expansion strategies toward rapidly developing emerging market economies led by the BRICS (Jakovljevic et al., 2016b). Probably a crucial part for the success rate of the global alcohol beverage industry lies in its devotion to specific focused advertising strategies (Wallack and Montgomery, 1992). It is a well-known fact that major competitors spend some 15% on marketing while approximately only 10% or slightly more on employee payroll expenses. Although, in many of these major companies net profit margin may be up to 25%. The mirror like reflection of this economic activity are revenues gained by the national governments worldwide which come from two sources: taxation on beverage manufacturers and value added tax or excise duties paid by the final consumers (Li and Si, 2016). An exceptionally important feature of global alcohol market is the fact that only the 38%-point share counts for branded beverages. The so called informal manufacturing, such as the rural beer brewing by Sudanese African women or traditional Eastern European distillation of a variety of local brandies, counts for a lion part of consumption worldwide (Jernigan, 2009). These traditional agricultural crafts are quite difficult to target by policy measures unlike major multinational businesses. Companies' irresponsible behavior aimed at increasing profits was documented in a number of publications (Casswell et al., 2016). Nonetheless, companies are accessible by evolving legal framework, imposing trade barriers, taxation measures and advertising bans which are frequently cited among the most cost-effective policy measures (Anderson et al., 2009).

There are visible signs of industrial attempt to adapt to growing public awareness on harmful effects of alcohol beverage advertising and expanding volume of sales. These businesses responded in several different ways. One of them was their investment into basic, clinical and social research on the general topic of alcohol (Babor, 2009), which raised strong concerns about the integrity of such science and conflict of interest issues due to the fact that producers are acting as major sponsors of grant holders (Stenius and Babor, 2010). Another approach was to establish The International Alliance for Responsible Drinking (IARD) while most major producers act as institutional affiliated members (International Alliance for Responsible Drinking n.d.). Its goal and core agenda was declared to tackle the global burden of harmful drinking by the citizens as final consumers. This issue is clearly reflected in the Sustainable Development Goals (GBD 2015 SDG Collaborators, 2016) and WHO's Global Strategy to Reduce the Harmful Use of Alcohol (World Health Organization, 2010). After a prolonged debate among the stakeholders dominant opinion among the experts is that alcohol beverage industry should not be involved in alcohol policy development. Exploration of both the regulatory and educational approaches in controlling alcohol consumption and sales has shown that the first one is by far more effective.

## Social cost of alcohol consumption

The Global Burden of Disease Project provided an in-depth and comparable morbidity and mortality assessments (Global Burden of Disease Cancer Collaboration et al., 2017) for all major world regions. It has clearly pointed out the contribution of alcohol to disability (GBD 2015 Disease Injury Incidence Prevalence Collaborators, 2016) and longevity. In 2015 alcohol use with 85 million global disability adjusted life years (DALYs) was still among the 10 largest contributors to global DALYs, although a decrease of 1.2% was noted since 2005 (Forouzanfar et al., 2016). In European countries a huge variation of years of life lost attributable to alcohol use exists, with almost ten-fold difference between highest and lowest rated country (Rehm et al., 2007). The connection between alcohol consumption and shorter life expectancy was probably the most exemplary in Russian mortality crisis of the 1990s (Andreev et al., 2003; Jakovljevic et al., 2016a).

Harmful use of alcohol imposes significant social and economic costs on society. Alcohol-attributable cost per head may range from I$358 to I$837 in high-income countries and from I$122 to I$524 in middle-income countries (Rehm et al., 2009). Indirect costs due to loss of productivity are the dominant category of costs which accounts about 49–95% of total costs, whereas direct costs encompass multiple types of health-care services representing about 9–24% of all alcohol-attributable social costs and significant costs in the justice sector as well (Rehm et al., 2009; World Health Organization, 2014).

Health authorities in a variety of countries became increasingly aware of the necessity to create strategies aimed to contain alcohol consumption at a certain level (Morgan, 1988). Obviously due to the scale of public health consequences, in some countries this issue was more concerning than others (Tulchinsky and Varavikova, 1996). A variety of policies deployed had rather modest or even a neglected impact to the sales and consumption of alcohol in many countries, particularly in the youth and adolescent population (Foster et al., 2006; Anderson et al., 2009).

Published evidence has clearly shown that regular consumption of spirits, beer, wine, or cider, presents a core risk factor for the occurrence of major non-communicable diseases (Forouzanfar et al., 2016). Clinical evolution of alcohol-attributable illnesses has been thoroughly studied and was the field of extensive medical knowledge development. It led to the rise of several distinguished classification of stages of alcohol addiction and abuse such as Clonninger, Babor, LAT and few others. Some of these, like LAT typology and its associated clinical software based guidelines were even documented to be capable of inducing cost savings in large university hospital settings (Jakovljevic et al., 2014b). Distinctively different cultural patterns of alcohol abuse have created a myriad of local conditions whose diversity in Europe has been well documented (Jakovljevic et al., 2013). A particularly concerning fact is that social cost of alcohol reached a scale that severely threatens financial sustainability of both Western and Asian health systems like the French one (Fenoglio et al., 2003), Canadian (Single et al., 1998), Californian (Rosen et al., 2008), Swedish (Jarl et al., 2008), New Zealand's' (Devlin et al., 1997), Indian (Benegal et al., 2000; Benegal, 2005) and many others. Preventive public health interventions were historically the first response option after policy makers awareness reached its maturity (Glasgow et al., 1999). However these strategies were either misunderstood or poorly supported by capacity building and resource allocation in many world regions (Room et al., 2002). Therefore curative, hospital based care for the medical consequences of neglected alcoholism became an option of choice for many communities (Jovanovic and Jakovljevic, 2011). This expensive, late stage solution raised the issue of affordable evidence based care of alcohol addicts both in psychiatric and internal medicine clinics (Jakovljevic et al., 2014a). Diagnostics, treatment and rehabilitation of alcohol dependent patients have proven to be major cost drivers (Jakovljević et al., 2013). Once again a variety of cost-effectiveness estimation efforts were put in place to rationalize resource allocation in this domain (Anderson et al., 2009). And once again strategic thinkers were more convinced that a long term measures targeted to make alcohol beverages less accessible to the ordinary citizen were probably the best solution to release this burden (Baumberg and Anderson, 2008). Reducing the affordability of alcohol by raising prices seems to lead to a reduction in alcohol consumption and associated harms—one of the successful policies is so called Minimum Unit Pricing, which consists of defining a level below which retailers cannot sell alcohol depending on the number of units per beverage (Institute of Alcohol Studies n.d.). It has been shown that 1% increase in the price of alcohol may lead to 0.96% reduction in alcohol consumption for the overall population of drinkers (Byrnes et al., 2016). However, it should be noted that rather than simply lowering consumed quantity in response to price increase, drinkers may as well be willing to switch to lower-cost brand in order to maintain their consumption (Gruenewald et al., 2006).

## Data report methods

Official data released by World Health Organization's Global Health Observatory Data Repository (European Region and broader (Russian) Commonwealth of Independent States observed) were downloaded from the links provided beneath: http://apps.who.int/gho/data/node.main-euro.A1113?lang=en&showonly=GISAH (World Health Organization n.d.). The data refer to the several key indicators of alcohol beverage industry revenues such as: Alcoholic beverage tax revenue as a per cent of government revenue; Annual revenues from alcohol excise tax in millions US$; Alcohol expenditure as a per cent of total household expenditure; Revenues from taxes on consumption (excise duties and similar charges) other than Value Added Tax (VAT)—ethyl alcohol in millions EUR; Revenues from taxes on consumption (excise duties and similar charges) other than VAT—intermediate products in millions EUR; Revenues from taxes on consumption (excise duties and similar charges) other than VAT—still wine in millions EUR. Extraction of selected data from this public repository is provided in Tables 1, 2. Another source of data was the European Commission's Taxation and customs union, Legislation section on Excise duties—Alcoholic beverages: http://ec.europa.eu/taxation_customs/business/excise-duties-alcohol-tobacco-energy/excise-duties-alcohol/excise-duties-alcoholic-beverages_en (European Commission n.d.). Data were synthesized in a comparable manner with emphasis on first and last historically reported value to the transnational authorities such as WHO and European Commission for European Union countries. Incremental gains and losses were calculated as annual and total net changes. Largest possible time horizon available was adopted and it ranged from 6 to 23 years on the set of total of 69 countries of Europe and broader CIS Region. There was a large amount of missing data and therefore conclusions refer to a limited set of countries providing sufficiently detailed data for comparison. Extraction and synthesis of aforementioned public registries on alcohol is archived as an excel sheet at FigShare link: https://figshare.com/articles/Alcohol_beverage_household_expenditure_taxation_and_government_revenues_in_European_and_CIS_countries/4245296.

**Table 1:** **Alcohol beverage taxation and government revenues—general**.

**Country**	**Alcoholic beverage tax revenue as a per cent of government revenue**					**Annual revenues from alcohol excise tax in millions US$**					**Alcohol expenditure as a per cent of total household expenditure**				
	**First reported value**	**Last reported value**	**Total change**	**Time span (years)**	**Annual increment**	**First reported value**	**Last reported value**	**Total change**	**Time span (years)**	**Annual increment**	**First reported value**	**Last reported value**	**Total change**	**Time span (years)**	**Annual increment**
United Kingdom	2.8^1990^	2.2^2013^	−0.6	23	−0.03	7,722.12^1991^	16,110.17^2013^	8,388.05	22	381.28	6.5^1990^	1.6^2012^	−4.9	22	−0.22
France	1.8^1990^	1.5^2007^	−0.3	17	−0.02	2,573.1^2002^	5706.25^2013^	3,133.15	11	284.83	1.4^2006^	N/A	N/A	N/A	N/A
Poland	N/A	N/A	N/A	N/A	N/A	2,228.88^2005^	3,271.75^2012^	1,042.87	7	148.98	8.5^1990^	0.8^2012^	−7.7	22	−0.35
Norway	2.1^1996^	1^2012^	−1.1	16	−0.07	869.02^1990^	3,243.53^2011^	2,374.51	21	113.07	4.6^1990^	2.6^2011^	−2	21	−0.10
Turkey	N/A	N/A	N/A	N/A	N/A	1,060.78^2003^	1,557.91^2010^	497.13	7	71.02	N/A	N/A	N/A	N/A	N/A
Sweden	1.8^1996^	1.6^2012^	−0.2	16	−0.01	1,206.96^2002^	1,794.99^2012^	588.03	10	58.80	3.7^1992^	2.8^2012^	−0.9	20	−0.05
Finland	8.4^1990^	5^2012^	−3.4	22	−0.15	1,063.39^1999^	1,739.81^2012^	676.42	13	52.03	7^1991^	4.4^2012^	−2.6	21	−0.12
Italy	0.6^1996^	N/A	N/A	N/A	N/A	751.58^2002^	1,249.95^2012^	498.37	10	49.84	1.1^1990^	0.7^2010^	−0.4	20	−0.02
Netherlands	1^1996^	N/A	N/A	N/A	N/A	970.2^2002^	1,273.44^2012^	303.24	10	30.32	N/A	N/A	N/A	N/A	N/A
Spain	1.8^1996^	N/A	N/A	N/A	N/A	1,160.62^2002^	1,444.94^2012^	284.32	10	28.43	1.3^1992^	N/A	N/A	N/A	N/A
Czech Republic	N/A	N/A	N/A	N/A	N/A	380.25^2005^	566.65^2012^	186.4	7	26.63	N/A	N/A	N/A	N/A	N/A
Greece	N/A	N/A	N/A	N/A	N/A	271.01^2002^	511.51^2012^	240.5	10	24.05	0.9^2000^	0.9^2009^	0	9	0.00
Belgium	1.1^1996^	N/A	N/A	N/A	N/A	533.69^2000^	799.49^2012^	265.8	12	22.15	N/A	N/A	N/A	N/A	N/A
Ireland	5^1996^	1.7^2010^	−3.3	14	−0.24	761.83^1994^	1,066.01^2012^	304.18	18	16.90	6^2000^	7.7^2012^	1.7	12	0.14
Estonia	10^1995^	3^2012^	−7	17	−0.41	59.83^2000^	277.45^2013^	217.62	13	16.74	1.7^1996^	3^2012^	1.3	16	0.08
Lithuania	N/A	N/A	N/A	N/A	N/A	206.86^2005^	314.04^2012^	107.18	7	15.31	6.1^1990^	2.4^2012^	−3.7	22	−0.17
Slovakia	N/A	N/A	N/A	N/A	N/A	215.24^2005^	321.4^2012^	106.16	7	15.17	N/A	N/A	N/A	N/A	N/A
Bulgaria	N/A	N/A	N/A	N/A	N/A	101.45^2006^	171.63^2012^	70.18	6	11.70	N/A	N/A	N/A	N/A	N/A
Latvia	N/A	N/A	N/A	N/A	N/A	114.792^2005^	182.01^2012^	67.218	7	9.60	2^1994^	N/A	N/A	N/A	N/A
Slovenia	N/A	N/A	N/A	N/A	N/A	75.33^2005^	117.1^2012^	41.77	7	5.97	N/A	N/A	N/A	N/A	N/A
Switzerland	0.5^1996^	N/A	N/A	N/A	N/A	244.38^2007^	278.5^2013^	34.12	6	5.69	N/A	N/A	N/A	N/A	N/A
Denmark	1.4^1996^	0.3^2012^	−1.1	16	−0.07	583.33^2002^	613.63^2012^	30.3	10	3.03	2.4^1994^	1.1^2012^	−1.3	18	−0.07
Hungary	N/A	N/A	N/A	N/A	N/A	339.59^2005^	360.44^2012^	20.85	7	2.98	1.1^2006^	1^2012^	−0.1	6	−0.02
Cyprus	N/A	N/A	N/A	N/A	N/A	25.11^2005^	34.89^2012^	9.78	7	1.40	0.6^1991^	N/A	N/A	N/A	N/A
Luxembourg	0.6^1996^	N/A	N/A	N/A	N/A	32.24^2002^	41.53^2012^	9.29	10	0.93	N/A	N/A	N/A	N/A	N/A
Malta	N/A	N/A	N/A	N/A	N/A	11.96^2005^	14.82^2012^	2.86	7	0.41	N/A	N/A	N/A	N/A	N/A
Portugal	0.9^1996^	N/A	N/A	N/A	N/A	215.6^2002^	211.51^2012^	−4.09	10	−0.41	N/A	N/A	N/A	N/A	N/A
Germany	1.7^1996^	0.6^2013^	−1.1	17	−0.06	3,433.49^2002^	3,414.33^2013^	−19.16	11	−1.74	N/A	N/A	N/A	N/A	N/A
Romania	N/A	N/A	N/A	N/A	N/A	331.04^2007^	298.37^2012^	−32.67	5	−6.53	11^1991^	0.4^2013^	−10.6	22	−0.48
Iceland	2.4^2003^	2.2^2012^	−0.2	9	−0.02	182.98^2007^	131.88^2012^	−51.1	5	−10.22	3.4^1990^	2.9^2007^	−0.5	17	−0.03
Austria	1^1996^	0.3^2010^	−0.7	14	−0.05	647.09^2005^	403.55^2012^	−243.54	7	−34.79	1.7^1995^	1.4^2013^	−0.3	18	−0.02
Belarus	N/A	N/A	N/A	N/A	N/A	N/A	N/A	N/A	N/A	N/A	7.4^1990^	2.5^2013^	−4.9	23	−0.21
Republic of Moldova	N/A	N/A	N/A	N/A	N/A	N/A	N/A	N/A	N/A	N/A	2.6^1990^	1.9^1995^	−0.7	5	−0.14
Russian Federation	N/A	N/A	N/A	N/A	N/A	N/A	N/A	N/A	N/A	N/A	5^1990^	1.7^2012^	−3.3	22	−0.15
Ukraine	N/A	N/A	N/A	N/A	N/A	N/A	N/A	N/A	N/A	N/A	3.7^1990^	1.5^2012^	−2.2	22	−0.10
Croatia	N/A	N/A	N/A	N/A	N/A	37.91^2008^	N/A	N/A	N/A	N/A	1.6^2005^	1.5^2011^	−0.1	6	−0.02
The former Yugoslav Republic of Macedonia	N/A	N/A	N/A	N/A	N/A	24^2007^	N/A	N/A	N/A	N/A	1.3^2006^	1.2^2011^	−0.1	5	−0.02
Armenia	N/A	N/A	N/A	N/A	N/A	N/A	N/A	N/A	N/A	N/A	4^1990^	1.5^2012^	−2.5	22	−0.11
Azerbaijan	N/A	N/A	N/A	N/A	N/A	N/A	N/A	N/A	N/A	N/A	1.3^1990^	0.5^2012^	−0.8	22	−0.04
Georgia	N/A	N/A	N/A	N/A	N/A	N/A	N/A	N/A	N/A	N/A	1.3^1990^	1.1^1991^	−0.2	1	−0.20
Kazakhstan	N/A	N/A	N/A	N/A	N/A	N/A	N/A	N/A	N/A	N/A	5^1990^	2.5^1995^	−2.5	5	−0.50
Kyrgyzstan	N/A	N/A	N/A	N/A	N/A	N/A	N/A	N/A	N/A	N/A	3.9^1990^	1.9^1995^	−2	5	−0.40
Tajikistan	N/A	N/A	N/A	N/A	N/A	N/A	N/A	N/A	N/A	N/A	1.8^1990^	0.1^2011^	−1.7	21	−0.08
Turkmenistan	N/A	N/A	N/A	N/A	N/A	N/A	N/A	N/A	N/A	N/A	3.4^1990^	4^1994^	0.6	4	0.15
Republic of Uzbekistan	N/A	N/A	N/A	N/A	N/A	N/A	N/A	N/A	N/A	N/A	2.9^1990^	1.9^1995^	−1	5	−0.20

**Table 2:** **Alcohol beverage taxation and government revenues—specific**.

**Country**	**Revenues from taxes on consumption (excise duties and similar charges) other than VAT—ethyl alcohol in millions EUR**					**Revenues from taxes on consumption (excise duties and similar charges) other than VAT—intermediate products in millions EUR**					**Revenues from taxes on consumption (excise duties and similar charges) other than VAT—still wine in millions EUR**				
	**First reported value**	**Last reported value**	**Total change**	**Time span (years)**	**Annual increment**	**First reported value**	**Last reported value**	**Total change**	**Time span (years)**	**Annual increment**	**First reported value**	**Last reported value**	**Total change**	**Time span (years)**	**Annual increment**
United Kingdom	3,357.62^2008^	3,928.21^2014^	570.59	6	95.10	326.45^2008^	415.38^2014^	88.93	6	14.82	3,226.04^2008^	4,368.1^2014^	1,142.06	6	190.34
France	2,001^2008^	2,239.47^2014^	238.47	6	39.75	106^2008^	76.15^2014^	−29.85	6	−4.98	85^2008^	90.38^2014^	5.38	6	0.90
Belgium	230.05^2008^	291.58^2014^	61.53	6	10.26	27.6^2008^	25.54^2014^	−2.06	6	−0.34	114.05^2008^	139.5^2014^	25.45	6	4.24
Austria	123.98^2008^	171.6^2014^	47.62	6	7.94	1.31^2008^	5.7^2014^	4.39	6	0.73	0^2008^	0^2014^	0	6	0.00
Croatia	22.59^2013^	30.37^2014^	7.78	1	7.78	N/A	N/A	*N*/*A*	N/A	N/A	0^2013^	0^2014^	0	1	0.00
Italy	554.67^2008^	600.26^2014^	45.59	6	7.60	N/A	N/A	*N*/*A*	N/A	N/A	0^2008^	0^2014^	0	6	0.00
Estonia	109.86^2008^	144.1^2014^	34.24	6	5.71	0.96^2008^	1.34^2014^	0.38	6	0.06	14.12^2008^	17.32^2014^	3.2	6	0.53
Bulgaria	66.08^2008^	95.74^2014^	29.66	6	4.94	0.0012^2008^	0.172^2014^	0.1708	6	0.03	0^2008^	0^2014^	0	6	0.00
Greece	257.46^2008^	282.31^2014^	24.85	6	4.14	N/A	N/A	*N*/*A*	N/A	N/A	0^2008^	0^2014^	0	6	0.00
Finland	415.35^2008^	430.11^2014^	14.76	6	2.46	16.84^2008^	17.7^2014^	0.86	6	0.14	227.95^2008^	336.18^2014^	108.23	6	18.04
Luxembourg	26.94^2008^	35.76^2014^	8.82	6	1.47	1.1^2008^	0.98^2014^	−0.12	6	−0.02	0^2008^	0^2014^	0	6	0.00
Slovenia	15^2008^	21.33^2014^	6.33	6	1.06	0.09^2008^	0.16^2014^	0.07	6	0.01	0^2008^	0^2014^	0	6	0.00
Cyprus	17.38^2008^	20.96^2014^	3.58	6	0.60	0.15^2008^	0.16^2014^	0.01	6	0.00	0^2008^	0^2014^	0	6	0.00
Portugal	92.8^2008^	96.11^2014^	3.31	6	0.55	12.95^2008^	10.99^2014^	−1.96	6	−0.33	0^2008^	0^2014^	0	6	0.00
Malta	9.786^2008^	11.426^2014^	1.64	6	0.27	N/A	N/A	*N*/*A*	N/A	N/A	0^2008^	0^2014^	0	6	0.00
Slovakia	199.9^2008^	200.314^2014^	0.414	6	0.07	0.404^2014^	N/A	*N*/*A*	N/A	N/A	0^2008^	0^2014^	0	6	0.00
Denmark	156^2008^	154.82^2014^	−1.18	6	−0.20	3.05^2008^	3.94^2014^	0.89	6	0.15	133.54^2008^	212.04^2014^	78.5	6	13.08
Hungary	182.69^2008^	175.07^2014^	−7.62	6	−1.27	2.55^2008^	1.19^2014^	−1.36	6	−0.23	0.46^2008^	2.1^2014^	1.64	6	0.27
Romania	98.65^2008^	88.98^2014^	−9.67	6	−1.61	29.6^2008^	0.38^2014^	−29.22	6	−4.87	0^2008^	0.26^2014^	0.26	6	0.04
Latvia	117.56^2008^	107.63^2014^	−9.93	6	−1.66	2.07^2008^	2.92^2014^	0.85	6	0.14	7.44^2008^	10.07^2014^	2.63	6	0.44
Sweden	441.63^2008^	430.09^2014^	−11.54	6	−1.92	20.32^2008^	16.65^2014^	−3.67	6	−0.61	413.95^2008^	532.14^2014^	118.19	6	19.70
Netherlands	338.94^2008^	322.09^2014^	−16.85	6	−2.81	37.49^2008^	26.75^2014^	−10.74	6	−1.79	239.07^2008^	319.19^2014^	80.12	6	13.35
Czech Republic	268.64^2008^	245.37^2014^	−23.27	6	−3.88	2.71^2008^	1.06^2014^	−1.65	6	−0.28	N/A	N/A	N/A	N/A	N/A
Lithuania	218.88^2008^	173.16^2014^	−45.72	6	−7.62	13.77^2008^	15.13^2014^	1.36	6	0.23	12.42^2008^	17.75^2014^	5.33	6	0.89
Ireland	350.92^2008^	301.78^2014^	−49.14	6	−8.19	66.46^2008^	67.33^2014^	0.87	6	0.15	213.25^2008^	332.78^2014^	119.53	6	19.92
Germany	2,125.93^2008^	2,059.66^2014^	−66.27	6	−11.05	27.11^2008^	14.67^2014^	−12.44	6	−2.07	0^2008^	0^2014^	0	6	0.00
Poland	1,632.94^2008^	1,536.27^2014^	−96.67	6	−16.11	N/A	N/A	N/A	N/A	N/A	126.26^2008^	86.2^2014^	−40.06	6	−6.68
Spain	981.82^2008^	826.9^2014^	−154.92	6	−25.82	19.96^2008^	19.56^2014^	−0.4	6	−0.07	N/A	N/A	N/A	N/A	N/A

## Alcohol beverage taxation and government revenues in the european region and broader commonwealth of independent states

Natural response of the national authorities to the huge medical and socioeconomic burden of alcohol use was a tightened control over its manufacturing and distribution (Levine and Reinarman, 1991). Many nations adopted the strategy targeted at limiting access to alcoholic drinks by making them effectively more expensive to final consumer. Among a variety of polices developed to tackle these issues we decided to observe taxation approach and consecutive changes in governmental/budgetary revenues.

We may notice several distinct trends taking place in Europe over the past two and a half decades. Household expenditure on alcohol expressed in percentage terms, was slightly growing since the early 2000s in only three countries with Ireland on the lead (+1.7%). Most data on this indicator come either from the Russian Commonwealth of Independent States (CIS) nations or post-2004 Eastern EU members. This share of family spending was steadily falling in all of these nations (Stroev et al., 1999). Surprisingly the most prominent decrease was noticed in Russian Federation, Lithuania, Belarus, UK, Poland in order of appearance with Romania topping the list with bottom—10.6% net change over 22 years. Due to tightened tax policies annual revenues from alcohol excise tax expressed in millions US$, grew significantly in most European nations. These amounts were exceptionally high in several large nations and Norway ranging from even + US$ 8,388.05 million, in the UK to only + US$ 2.86 million, in Malta. Only five countries recorded contraction of such income to the public budget and these were: Portugal, Germany, Romania, Iceland and Austria at the bottom with loss of even—US$ 243.54 million over 7 years. Unlike these amounts in absolute terms, in relative terms alcoholic beverage tax revenue contribution as a per cent of government revenues was steadily falling over the same period. This was effectively due to the stronger impact of other sources of budgetary revenues. Contraction of these revenues share was ranging from −7% in Estonia to −0.2% in Iceland. Total net increase in revenues from taxes on consumption such as excise duties and similar charges ^*^(other than VAT) recorded a bold growth upwards particularly in some Western European, EU-15 traditional free-market economies. This is clearly visible in ethyl alcohol, intermediary products, beer, sparkling wine and still wine official turnover and sales statistics. In all of these domains the UK is clearly dominating revenue growth. Probably the most notable example is their still wine income to public resources which surmounted to €1,142.06 million. The only major exception is beer industry and consumption in France whose taxation heavily increased putting France ahead of others. This meant + €560.63 million budgetary gains or almost €95 million annual growth over the 6 year time span 2008–2014. Some countries remained in certain sense outliers of this mainstream trend of harsher alcohol taxation polices in Europe. Their excise and similar taxes and consecutive national revenue rates remained rather constant and slowly decreasing over the past decade or more. This was the case with Portugal, Germany, Romania, Iceland and an extreme case of Austria who even lost −US$ 243.54 million, since 2005. If we observe the European landscape in revenues from taxes other than VAT, on ethyl alcohol we shall notice a number of countries with huge net losses. Bottom line ones are Lithuania, Ireland, Germany, Poland and Spain leading the picture with −€154.92 million of total losses since 2008.

Increase in annual revenues from alcohol taxes was also noted in the United States. Increase was substantial: from US$ 8.14 billion in 2000 to US$ 9.64 billion in 2015, with a forecast to continue in a similar manner to US$ 10.18 billion in 2021 (Statista n.d.). About 3% per cent of government revenue in the United States comes from excise tax on the sales of alcohol, tobacco and fuel (Center on Budget Policy Priorities, 2016).

## Alcohol beverage industry taxation policies in future

As witnessed by this short analysis of public datasets we may see that there has been a variety of strategies across the European region and broader Commonwealth of Independent States countries. We may see that a number of Western European EU-15 traditional free-market economies recorded tightened governments' grip on alcohol industry inclusive of double taxation of manufacturers, distributors and the final consumer. This example led by the UK obviously succeeded to gain huge net revenues into the public funds either at the regional or national level. Many other large countries such as Germany, Romania, and Spain did not adopt such policies. Their alcohol beverage taxation rates remained rather constant or even slightly contracted over past few decades. Due to large populations and alcohol consumption being deeply rooted as a part of popular culture and way of life, this led to the strong contraction of public revenues coming from this source of income. The case with the Russian Federation was marked with several positive developments (Rechel et al., 2013). Its household expenditure on alcohol severely shrank from 5% back in 1990 to only 1.7% in 2012 over the span of 22 years. At the same time rate of road traffic accidents involving alcohol per 100,000 inhabitants decreased from 47.18 in 1990 to 11.16 in 2009 over 19 years. Other not less effective polices were advertising bans on alcohol beverages deployed in some countries (Fieldgate et al., 2013). Investigation of the efficiency frontier and public health gains achieved with these measures goes well beyond the scope of this Data Report. Seeking at least partial answer we had insight into the road traffic accidents involving alcohol per 100,000 inhabitants presented in the original data (please see FigShare repositorium link) (Ruhm, 1996). Here it is clear that few nations managed to significantly reduce these rates with prominent cases of Denmark, Iceland, Russian Federation, Andorra and Luxembourg each cutting its own rates approximately for 30–45% since 1980s. Another angle of view could be taken while observing mortality issues—standardized death rates (SDR) attributable to the selected alcohol-related causes expressed per 100,000 inhabitants. These data are available for most countries since 1980s or even late 1970s. Here vast majority of nations made a huge advance with some of them decreasing these mortality rates for even more than 70% such as Spain, Turkmenistan, Italy, Hungary, Austria, Portugal, and France (Dee, 1999). Here it is worthy to mention few important outliers coming mostly from CIS region (Stickley et al., 2007). These are Kazakhstan, Belarus, Ukraine, Turkey, Tajikistan, and San Marino who all substantially increased their alcohol-attributable mortality with Kazakhstan on the lead with 84% in the 1991–2003. Due to the fact that some of these referral values are significantly outdated we should stay short of premature conclusions on contemporary momentum on alcohol in these regions (Shleifer and Treisman, 2005). Improved methods of the Global Health evaluation (Global Burden of Disease), strong epidemiologic evidence, improved knowledge of the alcohol harm bring together solid base for a change in Health policy toward the alcohol consumption. At the policy level, the hypothesis of health benefits from moderate drinking should no longer play a role in decision making (Chikritzhs et al., 2015). The number of nations across EU, CIS and intermediary regions, were decently successful in employing and persisting with alcohol industry taxation policies. The tendency to make the alcohol drinking habit expensive luxury good (Babor, 2010) in many countries appears to be quite effective distraction against heavy drinking patterns (Wilk et al., 1997). The long term consequences of these efforts remain yet to be seen in the upcoming years.

## Author contributions

MJ, EV, HW, AW, AP, OL all contributed essentially to the definition of research questions, study design and writing of the final manuscript thus fulfilling the ICMJE requirements for full authorship.

### Conflict of interest statement

The authors declare that the research was conducted in the absence of any commercial or financial relationships that could be construed as a potential conflict of interest.
